# Combined paralysis of the abducens and facial nerves following idiopathic intracranial hypertension

**DOI:** 10.1016/j.ijscr.2024.110071

**Published:** 2024-07-23

**Authors:** Kobra Sheidaee, Ali Abbaskhanian, Ali Mohammadi Kali, Fatemeh Rostamian Motlagh, Saeed Kargar-Soleimanbad

**Affiliations:** a Pediatric Infectious Diseases Research Center, Communicable Diseases Institute, Mazandaran University of medical Sciences, Sari, Iran; b Student Research Committee, Faculty of Medicine, Mazandaran University of medical Sciences, Sari, Iran; c Department of Neurology, Sari Medical Sciences Branch, Islamic Azad University, Sari, Iran

**Keywords:** Children, Idiopathic intracranial hypertension, VIth nerve, VIIth nerve, Pseudotumor cerebri

## Abstract

**Introduction:**

Idiopathic intracranial hypertension (IIH) is a clinical phenomenon that reflects an increase in intracranial pressure in the brain with normal parenchyma and no signs of ventriculomegaly, malignancy, infection, or any space-occupying lesion. Generally, this disease is associated with symptoms such as headache, transient visual obscurations (unilateral or bilateral darkening of the vision typically seconds), intracranial noise, diplopia, blurring of vision, abducens nerve palsies, and unilateral or bilateral facial nerve paresis (which is a very rare complication of this disease that has been reported in some studies).

**Case presentation:**

An 8-year-old boy with a history of bilateral frontal headache for 2 weeks, right ear pain, vomiting, and intermittent fever, who had received antibiotics and analgesics with improvement of ear pain and continuation of headache, presented to this center. In the initial neurological examinations, bilateral papilledema and right-sided 6th and 7th cranial nerve palsy (peripheral) were observed. After performing LP and CT scan and MRV for the patient, a diagnosis of pseudotumor cerebri was made and he was treated with acetazolamide, prednisolone, and topiramate. He was discharged after 10 days.

**Conclusion:**

Although pseudotumor cerebri is less common in children than adults and obesity and female gender are considered as risk factors for this disease, it is not usually associated with involvement of the 6th and 7th cranial nerves. However, sometimes this disease can occur in children without any risk factors and with less common involvement of the 6th and 7th cranial nerves.

## Highlights

• Idiopathic intracranial hypertension is a clinical phenomenon that reflects an increase in intracranial pressure.
• IIH is divided into two types based on the underlying cause.
• Sometimes IIH occur in children with involvement of the 6th and 7th cranial nerves.

## 1 Introduction

Idiopathic intracranial hypertension (IIH) or pseudotumor cerebri was first described by Quincke in 1897. IIH is a clinical phenomenon that reflects an increase in intracranial pressure in the brain with normal parenchyma and no signs of ventriculomegaly, malignancy, infection, or any space-occupying lesion. Generally, this disease is associated with symptoms such as headache, transient visual obscurations including unilateral or bilateral darkening of the vision typically seconds, intracranial noise, diplopia, blurring of vision, abducens nerve palsies, and unilateral or bilateral facial nerve paresis, which is a very rare complication of this disease that has been reported in some studies [1].

IIH is divided into two types based on the underlying cause: idiopathic and secondary [2]. Although the exact cause of idiopathic IIH is unknown, multiple etiologies have been proposed including meningitis, metabolic disorders and obesity, cerebral venous hypertension, anemia, obstructive sleep apnea, renal failure, Cushing's disease, Addison's disease, hypoparathyroidism, Turner syndrome, systemic lupus erythematosus, pharmacological factors such as antibiotics like tetracyclines and their derivatives, hyper-vitaminosis A and its derivatives such as isotretinoin and all-trans retinoic acid, corticosteroids, growth hormone, thyroxin replacement in children, and drugs such as nalidixic acid, lithium, rifampicin, cimetidine, and estrogen-progestin contraceptives for secondary IIH [3,4].

Diagnosis of this condition is usually made by ruling out other causes and considering all the criteria listed in Table 1 [5].

**Table 1:** Diagnostic Criteria for IIH.

Diagnostic criteria for IIH
Papilledema
Normal neurological examination except for sixth cranial nerve abnormalities
Neuroimaging: Normal brain parenchyma without evidence of hydrocephalus, mass or structural lesion, and no abnormal meningeal enhancement on MRI, with and without gadolinium, for typical patients (female and obese), and MRI, with and without gadolinium, and magnetic resonance venography for others; if MRI is unavailable or Contraindicated, contrast-enhanced CT may be used.

Normal CSF composition
Elevated lumbar puncture opening pressure (≥250 mm CSF in adults, ≥280 in children) in a properly performed lumbar puncture

While IIH is more common in obese women of childbearing age between 20 and 40 years old, affecting approximately 90 % of patients who are women with a prevalence of 1–3 cases per 100,000 people, it can also occur in children with a prevalence of 0.1–0.9 cases per 100,000 people, with equal incidence in both sexes [6,7]. In addition to causing headaches and disrupting normal life, IIH can also cause other complications, with vision loss being the worst. However, with timely diagnosis and treatment, this complication can be prevented [8].

We report the case of an 8-year-old boy who presented with combined VIth and ipsilateral VIIth nerve palsy following IIH.

## 2 Case presentation

An 8-year-old boy presented with a 2-week history of bilateral frontal headache, right ear pain, vomiting, and intermittent fever. He was treated with antibiotics (co-amoxiclav and cefixime) and analgesics (paracetamol and ibuprofen) by an ENT specialist, which improved his ear pain but not his headache, which progressed to involve the entire head. One day before hospital admission, he developed diplopia, photophobia, vomiting, and right facial paralysis. He had no relevant past medical history except for unilateral kidney on prenatal ultrasound. He was not taking any long-term medications. There was no family history of neurological disease except for his sister's diagnosis of Fosse syndrome. On examination, he had bilateral papilledema, bilateral lower limb edema, and right-sided VIth and ipsilateral VIIth nerve palsy. His brain CT was normal, and he was treated with ceftriaxone, vancomycin, and acyclovir. Subsequent brain MRI with gadolinium and MRV were also normal. Lumbar puncture performed,

Laboratory tests on the CSF, including analysis, smear preparation, culture, and infectious tests, did not yield any pathological results (Table 2). Opening pressure in LP was 430 mmH2O, which decreased to 380 mmH2O after treatment with acetazolamide and prednisolone. After 10 days of hospitalization, he was discharged on prednisolone 50 mg, topiramate 50 mg, acetazolamide 250 mg, vitamin B6 40 mg, and folic acid tablets. Three months later, his VIIth nerve palsy had resolved completely, and he had no specific complaints. He was also negative for COVID-19 on CT scans, RT-PCR, and serology tests. Current study has been reported in line with the SCARE criteria [9].

**Table 2:** Laboratory findings.

Laboratory tests	
WBC	7600
RBC	4,060,000
HGB	12.8
HCT	35.3 %
MCV	86.9
MCH	31.5
MCHC	36.5
PLT	296,000
LYM	28.2
NEUT	61.5
MXD	10.3 %
CSF/C	Neg

## 3 Discussion

We report a case of an 8-year-old boy with weight loss, prolonged headache, photophobia, diplopia, bilateral papilledema, and simultaneous paralysis of the right abducens and facial nerves caused by idiopathic intracranial hypertension (IIH). Although MRI was normal in this patient despite high intracranial pressure, IIH with pseudotumor cerebri was considered as the primary suspicion due to the absence of any signs of CNS involvement and normal neurological examinations in cranial nerves except for the 6th and 7th pairs. After performing a lumbar puncture, the diagnosis of pseudotumor cerebri was confirmed.

IIH in children is a rare finding compared to adults (0.1–0.9 vs. 1–3 per 1,000,000), but its occurrence in prepubertal children is very uncommon [10]. Although the main pathogenesis of this disease is not clear, theories suggest that factors leading to increased CSF production, decreased CSF absorption by arachnoid villi, and impedance to venous return from the brain are the main causes of this condition [11]. While female gender and obesity are introduced as effective risk factors for IIH in adults (6:1 female-to-male ratio), studies on these risk factors in children under 18 years old are not very consistent. Some studies, including Balcer et al., reported that 43 % of pediatric IIH patients were obese, weighing >120 % of their peers' ideal weight. Another study by Kesler et al. found that 23 % of adolescent IIH patients had a BMI above 30. However, in our reported case, the patient was underweight and did not exhibit weight gain [12,13].

Cranial nerve palsy is observed in some cases of IIH in children, especially in prepubertal children. Among them, the involvement of the abducens nerve is the most common, with a prevalence rate between 10 and 20 % in different studies [14,15]. The next most common cranial nerve involvement after abducens is related to the facial nerve, which is reported in 2–6 % of pediatric patients with IIH [16]. Although involvement of other cranial nerves such as trigeminal, trochlear, oculomotor, glossopharyngeal, and hypoglossal nerves is rare, it has been reported. Usually, involvement of only one cranial nerve is not a rare event in IIH, but the simultaneous involvement of two nerves is considered a very rare finding in these patients [14,17].

Visual impairment in IIH can be a complaint and a sign of the disease, and if left untreated, it can become a tragic complication for patients. In Rangwala et al.'s study, IIH complications in pediatric patients included visual field loss in 90 % of cases and visual acuity loss in 6–20 % of cases [18]. Therefore, timely treatment and rapid intervention for these patients are essential, both pharmacologically and surgically if necessary. Even after treatment, evaluation of vision in these patients is recommended [18]. The first step in treating these patients is to reduce intracranial pressure by any means, such as prescribing steroids or acetazolamide or a combination of both, which can reduce symptoms and prevent the progression of vision loss [19].

In Soroken et al.'s study, which investigated cases of pediatric patients with simultaneous involvement of the 6th and 7th cranial nerves, only four cases were reported. Among them, the cases that were female and post-pubertal were observed, and only one case of an 8-year-old boy with unilateral involvement of both the 6th and 7th nerves was observed [20,21]. The case we reported is a very rare phenomenon of an 8-year-old prepubertal boy with weight loss who had simultaneous unilateral involvement of both the 6th and 7th cranial nerves and can be considered a unique case.

## 4 Conclusion

Although IIH or pseudotumor cerebri, which is known as idiopathic intracranial hypertension, is a less common disease in children than adults and is associated with obesity and female gender as risk factors, we reported a case of an underweight 8-year-old boy with simultaneous involvement of both VIth and VIIth cranial nerves. Despite the rarity of this phenomenon, the patient responded to treatment with prednisolone, topiramate, and acetazolamide after two LPs, and his symptoms resolved after some time. However, pseudotumor cerebri should be considered as a disease with severe and irreversible complications such as visual impairment, especially in patients who present with neurological symptoms but have only papilledema and involvement of the less common VIth and VIIth cranial nerves on MRI and neurological examination. Early treatment can prevent these irreversible complications.

## Consent

Written informed consent was obtained from the patient for publication of this case report and accompanying images. A copy of the written consent is available for review by the Editor-in-Chief of this journal on request.

## Ethical approval

Considering national committee for ethics in biomedical research lows. It is not necessary to get Ethical Approval code for case reports, only patients Consent is enough.

## Funding

None.

## Author contribution

S·K involved in interpretation and collecting of data, and editing the manuscript. K·S, A.A, A.M and F.R involved in writing, editing and preparing the final version of manuscript. All authors reviewed the paper and approved the final version of the manuscript.

## Guarantor

Saeed kargar-soleimanabad.

## Research registration number

N/A.

## Conflict of interest statement

The authors report no conflicts of interest.
